# Bilateral Nephrectomy, the Forgotten Measure in the Treatment of Refractory Hypertension in Patients With End-Stage Renal Disease: A Case Report and Literature Review

**DOI:** 10.7759/cureus.9031

**Published:** 2020-07-06

**Authors:** Mohammad Aldiabat, Khaled Alabdallah, Ahmad Kofahi, Shazia Aziz

**Affiliations:** 1 Internal Medicine, Lincoln Medical Center, New York, USA

**Keywords:** end stage renal disease (esrd), bilateral nephrectomy, high blood pressure, hemodialysis, polycystic kidney disease, refractory hypertension, resistant hypertension

## Abstract

It's not uncommon for patients with end-stage renal disease (ESRD) to develop hypertension that is resistant to antihypertensive medications and volume control, making it a challenge to control blood pressure in those patients. In this article, we present a 71-year-old female with a history of ESRD on intermittent hemodialysis (IHD), who developed refractory hypertension despite the use of seven antihypertensive agents in addition to IHD. The patient underwent bilateral nephrectomy as a last resort therapy for managing resistant hypertension, which led to a significant improvement in blood pressure (BP) and decreasing the number and doses of antihypertensive agents. This article aims to raise the awareness and alertness of clinicians to the efficacy of bilateral nephrectomy as rescue therapy for refractory hypertension in hemodialysis patients.

## Introduction

Chronic kidney disease patients are at risk of developing hypertension secondary to volume overload, sympathetic over-activity, activation of the renin-angiotensin system, arteriosclerosis, and noncompliance with antihypertensive medications. Some patients with end-stage renal disease (ESRD) on intermittent hemodialysis (IHD) are resistant to volume control and antihypertensive medications for the treatment of hypertension. A diagnosis of resistant hypertension (HTN) is made in these patients when the patient's blood pressure (BP) is uncontrolled in spite of the concurrent use of three antihypertensive agents of different classes or if BP is controlled with four or more medications [1] as compared to refractory hypertension when BP is uncontrolled despite using five or more drugs, including chlorthalidone and a mineralocorticoid receptor antagonist under the care of a hypertension specialist [2].

In rare cases of refractory hypertension in ESRD patients whose elevated BP is resistant to all interventions, including IHD medications and hemodialysis, bilateral nephrectomy is considered an effective method for the management of refractory hypertension in hemodialysis patients among other invasive and experimental lines of treatment [3].

## Case presentation

**Figure 1:**
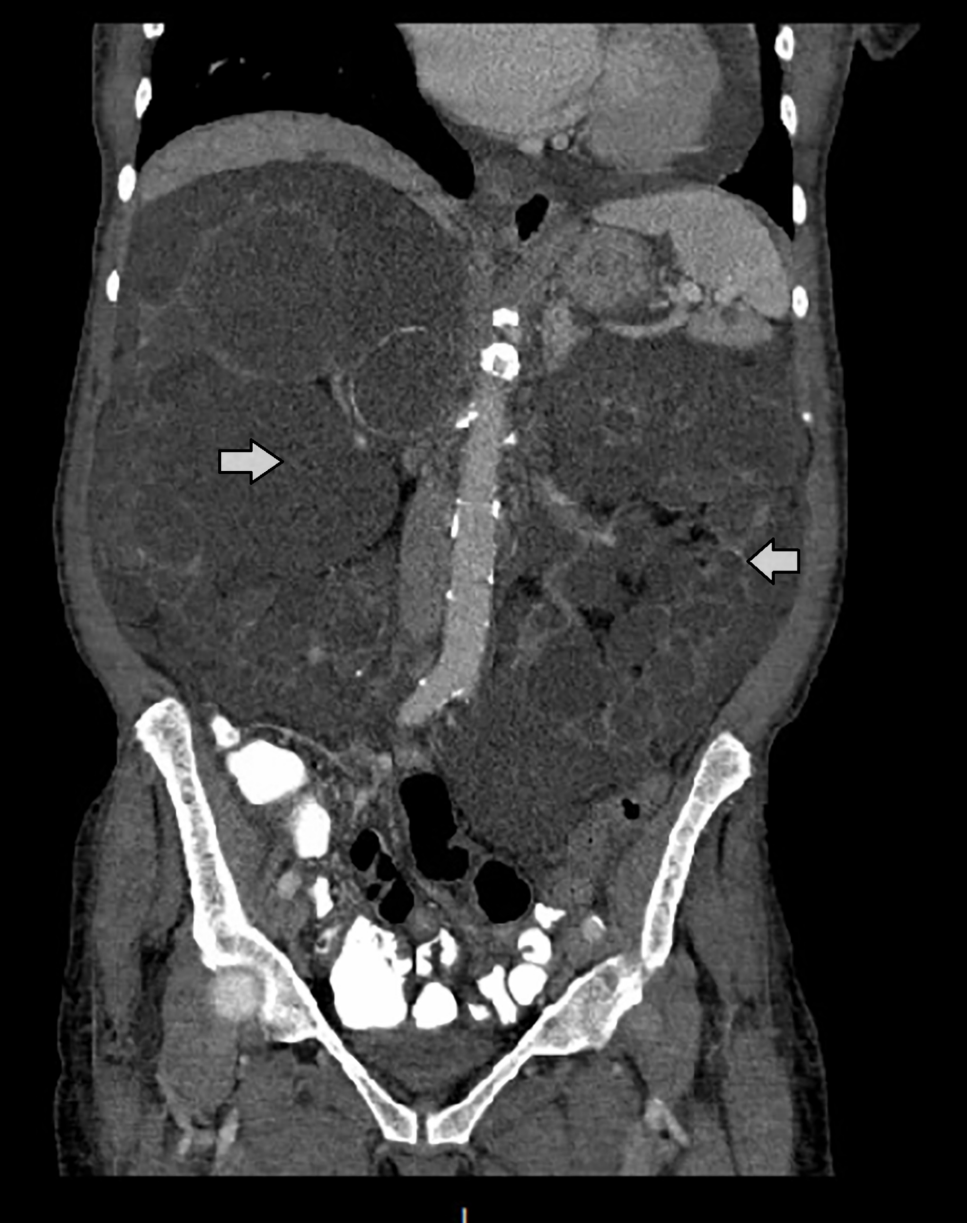
CT abdomen showing the severe cystic appearance of both kidneys (white arrows) CT: computed tomography

We present a 71-year-old female, with a past medical history of hypertension, hyperlipidemia, end-stage renal disease (secondary to polycystic kidney disease, Figure 1), on intermittent hemodialysis who was admitted to the hospital with a hypertensive emergency, with systolic blood pressure (SBP) > 200, complicated with non-ST-elevation myocardial infarction (NSTEMI) type 2, diastolic heart failure, pericardial effusion, hypertensive retinopathy, headache, and tinnitus. The patient's elevated BP was resistant to the oral antihypertensive regimen and daily hemodialysis sessions. The patient had a previous admission with a hypertensive emergency that resolved with multiple sessions of hemodialysis. The patient's antihypertensive regimen included clonidine 0.6 mg PO TID, hydralazine 100 mg PO BID, labetalol 800 mg BID, lisinopril 10 mg QD, nifedipine XL 240 QD, and spironolactone 100 mg QD. Minoxidil was relatively contraindicated in this patient given the current pericardial effusion. The patient's BP was controlled with the addition of intravenous (IV) nicardipine drip with the goal of SBP < 180 and was transferred to the critical care unit for close monitoring.

The secondary causes of resistant hypertension in this patient were investigated, including primary hyperaldosteronism (unlikely given that the patient's BP did not improve with spironolactone 100 mg QD), renal artery stenosis (unlikely as the patient was found to have mild focal stenosis of the left renal artery on magnetic resonance (MR) angiography in addition to mild to moderate spinal muscular atrophy (SMA) stenosis), age-associated arteriolosclerosis, pseudo-resistant hypertension (aortic blood pressure reading through left heart catheterization was compatible with peripheral readings), and medications noncompliance. The patient remained dependent on a nicardipine drip despite multiple regimen oral antihypertensives and daily four-hours hemodialysis sessions. Choices for the treatment of this patient's refractory hypertension were considered, including renal denervation and bilateral nephrectomy. Multiple disciplinary meetings of the patient's cardiologist, endocrinologist, nephrologist, and urologist were held and a decision was made to proceed with bilateral nephrectomy. The choice of renal denervation was deferred in this patient, as it’s an invasive procedure with unproven efficacy in the treatment of resistant hypertension.

Bilateral nephrectomy took place with no major complication of the patient except for the transfusion of three units of packed red blood cells (RBCs) due to intraoperative bleeding. The patient was monitored in the coronary care unit (CCU) for one week after the procedure, remained dependent on the nicardipine drip, which tapered down until discontinued on Day 8 postop, with the SBP range between 160 and 180. The patient's BP continued to normalize afterward, over a period of three months, with previous and current SBP ranges listed in Table 1. Lisinopril was discontinued after the surgery, labetalol was discontinued one month late, and the doses of the rest of the patient's antihypertensives were reduced gradually. Clinically, the patient's overall picture and quality of life improved, as symptoms of headache and tinnitus improved and the patient was weaned off the nicardipine drip. Afterward, the patient was started on erythropoietin replacement therapy to maintain effective erythropoiesis.

**Table 1:** Blood pressure reading prior to, one month after, and three months after bilateral nephrectomy SBP: systolic blood pressure

SBP Before over 1 month (lowest-highest)	SBP After Bilateral Nephrectomy (lowest-highest)	
	1 Month	3 Months
185/65-220/94	165/62-125/59	153/60-91/48

## Discussion

Resistant hypertension refers to elevated BP that is uncontrolled in spite of the concurrent use of three antihypertensive agents of different classes or if BP is controlled with four or more medications as compared to refractory hypertension when BP is uncontrolled despite using five or more drugs, including chlorthalidone and a mineralocorticoid receptor antagonist under the care of a hypertension specialist. Patients with resistant and refractory HTN are at risk of significant comorbid conditions, as one study showed that patients with resistant hypertension have a 32% increase in the risk of developing ESRD, a 24% increase in the risk of myocardial ischemia, a 46% increase in the risk of heart failure, a 14% increased risk of cerebrovascular events, and a 6% increased risk of mortality. Patients with chronic kidney disease are at risk of developing HTN, resistant HTN, and refractory HTN secondary to volume overload, sympathetic overactivity, activation of the renin-angiotensin system, and arteriosclerosis, with a prevalence of 50%-60% of ESRD patients on hemodialysis being hypertensive, and a prevalence of resistant hypertension of 33.4%, 28.9%, and 15.8% in chronic kidney disease with estimated glomerular filtration rate (eGFR) levels of <45 ml/min, 45-60 ml/min, >60 ml/min, respectively [1,3-4].

Other secondary causes of resistant hypertension should be investigated before attributing it to chronic kidney disease and before proceeding with invasive management, including primary aldosteronism (10%-20% of resistant hypertension), renal artery stenosis secondary to fibromuscular dysplasia or atherosclerosis in older patients, or obstructive sleep apnea, with one study showing a 71%-85% prevalence of obstructive sleep apnea (OSA) in patients with resistant hypertension referred to in sleep studies [5-8].

Controlling BP in patients with ESRD on dialysis decreases mortality secondary to cardiovascular events, as one study showed a decrease in cardiovascular events, mortality, and all-cause mortality in ESRD on dialysis and patients whose BP was controlled with antihypertensive therapy. After achieving optimal dry weight through dialysis by increasing dialysis time and decreasing dialysate sodium [9-10], antihypertensive medication lines of treatment include beta-blockers as first-line with atenolol and dihydropyridine calcium blockers as second-line treatment with amlodipine [11-12]. An angiotensin-converting enzyme (ACE) inhibitor or angiotensin receptor blocker (ARB) can be added later on if the earlier regimen is not effective. Adding minoxidil (in our patient, minoxidil was relatively contraindicated, as the patient was found to have pericardial effusion), spironolactone [13], and clonidine can be considered if no modifiable cause of resistant hypertension is present and medication noncompliance is ruled out.

If the above regimen is ineffective, more invasive measures can be considered such as bilateral nephrectomy. Other experimental methods, including renal denervation and stimulation of carotid sinus baroreceptors, is being studied. Bilateral nephrectomy is the most effective intervention in reducing blood pressure in patients with resistant/refractory hypertension to any other therapy [3]. It works by shutting down renin-angiotensin-aldosterone levels by removing the diseased kidneys where the renin is being secreted. In dialysis patients, it has been proposed that ultrafiltration of sodium can potentiate renin-angiotensin activation and worsen blood pressure [14]. Bilateral nephrectomy was used in the past for treating hypertension but it is overlooked now given the introduction of more effective antihypertensive agents. In one study where 10 patients with ESRD on dialysis underwent bilateral nephrectomy, a significant decrease in systolic and diastolic blood pressure was found before and after the surgery, with a mean SBP of 191 ± 20 vs 133 ± 18 and a diastolic blood pressure (DBP) of 116 ± 12 vs 82 ± 14 (p-value <0.005) [3], with improved clinical status and quality of life and from the financial point of view, as less blood pressure medications are needed. After surgery, patients will require supplementation with erythropoietin and serial complete blood count (CBC) monitoring to correct anemia.

Other invasive interventions for treating hypertension include renal denervation, by which catheter-based radiofrequency or ultrasound ablation of renal sympathetic nerves is done. Although this method is found effective in BP reduction in hypertensive patients, its role in treating resistant hypertension has not been established [15]. Experimental interventions for resistant hypertension include the stimulation of carotid sinus baroreceptors, which was not approved in the US after the Rheos Pivotal Trial failed in two out of five primary endpoints (efficacy and safety of using baroreflex activation therapy) [16-17] and central arteriovenous anastomosis, which, although found to cause a significant reduction in blood pressure, does need further studying before being recommended in the treatment of resistant hypertension [18].

## Conclusions

Among the invasive interventions in treating resistant hypertension in patients with end-stage renal disease on dialysis despite dietary and lifestyle modifications, including increased dialysis time, decreased sodium dialysate, and the administration of multiple antihypertensive agents, bilateral nephrectomy is the most effective measure for treating hypertension in terms of lowering blood pressure, improving the clinical picture and quality of life, and reducing the number and dosage of antihypertensive medications in these patients.
